# Exploring the educational challenges in emergency medical students: A qualitative study

**DOI:** 10.30476/jamp.2021.88535.1344

**Published:** 2021-04

**Authors:** ALI AFSHARI, MASOUD KHODAVEISI, EFAT SADEGHIAN

**Affiliations:** 1 Student Research Committee, Hamadan University of Medical Sciences, Hamadan, Iran; 2 Chronic Diseases (Home Care) Research Center, Department of Community Health Nursing, Hamadan University of Medical Sciences, Hamadan, Iran; 3 Chronic Diseases (Home Care) Research Center, Department of Nursing, Hamadan University of Medical Sciences, Hamadan, Iran

**Keywords:** Education, Emergency medical services, Qualitative study, Students

## Abstract

**Introduction:**

Emergency medical services (EMS) are provided in the difficult and unpredictable conditions of prehospital settings. Therefore, EMS students need to receive quality education to acquire considerable knowledge and skills. This qualitative study aimed to explore the educational challenges in medical emergency students.

**Methods:**

This qualitative study was carried out in 2019 using qualitative content analysis. Participants were fourteen undergraduate EMS students purposively recruited from Hamadan University of Medical Sciences, Hamadan, Iran. Data collection was done through semi-structured interviews and continued up to data saturation. Data were analyzed using the conventional content analysis approach explained by Graneheim and Lundman.

**Results:**

The four main categories of the challenges of EMS education were shortcomings of the clinical education environment, lack of qualified EMS instructors, deficit of the curriculum, and theory-practice gap.

**Conclusion:**

During their university education, EMS students face different challenges. For effective training, more coordination is needed among the emergency medical board, faculty members and EMS managers. It is also recommended that the curriculum should be revised.

## Introduction

The emergency medical services (EMS) system has a significant contribution to the management of emergency incidents and disasters ( 1 ). EMS technicians assess and manage the patients’ conditions, support their lives, and provide them with first aid services in prehospital settings. Besides, they have other responsibilities such as ambulance driving and EMS documentation ( 2 , 3 ).

EMS technicians’ knowledge, skills, and experience have significant effects on the patient outcomes and survival ( 4 ). Their wise and timely decisions can ensure the patients’ safety and health, facilitate their recovery, and reduce the risk of complications ( 5 ). A former study highlighted that appropriate education and adequate professional experience were the basic requirements for appropriate decision making in emergency situations and noted that EMS technicians should never use the trial-and-error method in their missions ( 6 ).

EMS students’ ae educated both theoretically and practically. In theoretical education, they receive education about basic sciences and specialized courses on prehospital EMS. In practical education, they develop their skills and experience through providing care to patients and victims in real prehospital and hospital settings. Given the significant roles of EMS in patient outcomes, the educational program of EMS students should improve not only their knowledge and practical skills, but also their communication and critical thinking skills ( 7 ).

Studies into EMS education in Iran were mostly conducted on EMS graduates. Two quantitative studies in this area reported that EMS graduates had inadequate professional competence and weak professional performance ( 8 , 9 ). A qualitative study into the perspectives and experiences of EMS graduates and instructors also reported different problems and challenges in EMS education, including inappropriate teaching methods, theory-practice gap, and incomprehensiveness of the EMS curriculum ( 10 ). Studies conducted on EMS graduates have suggested that more studies should be conducted on students. On the other hand, researchers, as teachers in EMS field, have witnessed the existence of educational problems among the EMS students. However, although students are a good source for identifying problems in education, our literature search showed that there was no study on the challenges of EMS education from their perspectives. Therefore, the present study was conducted to address this gap. The aim of the study was to explore the educational challenges in medical emergency EMS students. 

## Methods

This qualitative study was conducted using conventional content analysis which is a research method for the subjective interpretation of textual data through systematic coding and categorization ( 11 ). The study was conducted from May to October 2019.

### Participants

Participants were fourteen undergraduate EMS students in Hamadan University of Medical Sciences, Hamadan, Iran. Inclusion criteria were agreement for participation and studentship in prehospital EMS for at least one year. In other words, the participants were selected from the third and fourth-semester students who had received both theoretical and practical education. Sampling was done purposively with EMS students who met the inclusion criteria considering maximum variation sampling. 

### Data collection

For data collection, semi-structure interviews were held by the first author. Interview questions included, *“May you please explain about educational courses and learning activities at the university”*? *“May you please explain about clinical training courses and learning activities in the hospital and EMS stations?”* Then, according to the answers, the interview questions were directed towards the purpose of the study and the educational problems were explored. Also, follow-up questions such as *“What do you mean by this?”*, *“Can you explain more about this?”* and *“How did you feel then?”* were used for further clarification. Interviews were held in the participants’ preferred time and place and lasted 45–60 minutes. All interviews were recorded and transcribed verbatim.

### Data analysis

The conventional content analysis approach explained by Graneheim and Lundman was used for data analysis. The five steps of this approach are immediate transcription of each interview, reading interview transcript for understanding its main ideas, identifying meaning units and primary codes, categorizing similar codes, and identifying the latent content of the data ( 12 ). Immediately after each interview, it was transcribed using the Microsoft Word software and the transcript was read for several times. Then, meaning units were identified and coded, and the codes were grouped into categories according to their similarities. During data analysis, the categories were further developed, combined, refined, and grouped into larger categories.

### Trustworthiness

Guba and Lincoln’s criteria ( 13 ) were used to ensure the trustworthiness. Credibility was ensured through prolonged engagement with the data and member checking in such a way the transcripts were made available to participants along with the initial codes, and some of the codes were modified to clarify the meanings of the sentences. Confirmability was ensured through peer checking by three faculty members and they confirmed all the steps of data extraction and analysis. To ensure dependability, each of the research team members independently coded the data, and then they discussed their findings to reach agreement. Sampling with maximum variation and detailed description of the study methods were also used to ensure transferability.

Ethical considerations

The Ethics Committee of Hamadan University of Medical Sciences, Hamadan, Iran, provided ethical approval for the study (code: IR.UMSHA.REC.1397.906). Before interviews, the participants were informed about the study aim, voluntariness of participation, and their right to unilaterally withdraw from the study; they were also asked to sign the informed consent form of the study.

## Results

All participants were male with an age range of 25–34 and an average work experience of four years. Nine participants were married and five were single. Eight participants were in the third semester and six of them were fourth-semester students.

The participants’ experiences of the challenges of EMS education were grouped into four main categories, i.e. shortcomings of the clinical education environment, lack of qualified EMS instructors, deficit of the curriculum, and theory-practice gap (Table 1).

**Table 1:** The challenges of EMS education

Main categories	Subcategories
Shortcomings of the clinical education environment	Allocation of most practical courses to hospital environment
	Limited experience in real prehospital settings
	Performance of non-specialized tasks in practical courses
Lack of qualified EMS instructors	Clinical education by inexperienced instructors
	Use of non-specialized instructors for theoretical courses
Deficit of the curriculum	No use of modern teaching methods
	Incongruence learning outcomes and job description
Theory-practice gap	Inadequacy of specialized courses
	Incongruence between protocols and theoretical knowledge
	Non-adherence to care standards in prehospital settings

### 1. Shortcomings of the clinical education environment

The participants highlighted that their practical courses were not offered in appropriate educational environments. The three subcategories of this main category were allocation of most practical courses to hospital environment, limited experience in real prehospital settings, and performance of non-specialized tasks in practical courses.

#### 1.1. Allocation of most practical courses to hospital environment

The participants noted that although their future work would be in prehospital settings, most of their practical courses were offered in hospital wards.

*“I remember that we had two clinical courses with an interval in the internal medicine ward, where we had nothing to do except for checking vital sings and administering medications”* (P7).

#### 1.2. Limited experience in real prehospital settings

The participants were dissatisfied with having no opportunity to attend prehospital settings and develop their experience of prehospital emergency care delivery.

*“We will soon be graduated in several months, but we have been dispatched only on five prehospital missions. We have not experienced many things”* (P11).

#### 1.3. Performance of non-specialized tasks in practical courses

The participants referred to the performance of non-specialized tasks during their hospital courses as the main challenge of their practical education.

*“We were in the oncology ward. Our instructor asked us to write a care plan for patients based on the steps of the nursing process”* (P2).

### 2. Lack of qualified EMS instructors

According to the participants, one of the main challenges of EMS education is the lack of qualified instructors. Quality EMS education requires instructors with EMS background and adequate EMS-related knowledge, skills, and experience. The two subcategories of this category were using nursing instructors for clinical education and using non-specialized instructors for theoretical courses.

#### 2.1. Clinical education by inexperienced instructors

Some participants stated that most of their practical courses were taught by nursing instructors who had no experience of prehospital EMS delivery.

*“The most important place for our practical learning is where our practical courses are offered. However, our instructors in these courses are novice nurses who have limited knowledge and skills for EMS education”* (P8).

#### 2.2. Using non-specialized instructors for theoretical courses

The participants expected that instructors with previous experience in EMS teach them theoretical courses on trauma, triage, and patient transfer.

*“I have no motivation for listening to an instructor’s lecture if he/she has no work experience in prehospital EMS”* (P1).

### 3. Deficit of the curriculum 

Some participants noted that their academic curriculum had deficits and also the teaching methods were repetitive, boring, and teacher-centered. The three subcategories of this category were no use of modern teaching methods, incongruence between some courses and EMS technicians’ job description, and inadequacy of specialized courses.

#### 3.1. No use of modern teaching methods

Education for EMS students should be provided using modern teaching methods such as problem solving, simulation, and other methods which can improve their decision making and practical skills in the shortest possible duration.

*“Public universities should be the pioneers of modern teaching. However, the lecture method is used in almost all of our classes and we just listen to the instructors’ lectures”* (P9).

#### 3.2 Incongruence between learning outcomes and job description

Participants noted that some of their learning outcomes were incongruent with EMS technicians’ job descriptions and prehospital EMS delivery.

*“Some courses are not relevant to our job description at all. They taught materials which are not in our job description and are among the tasks of firefighters and relief and rescue staff”* (P11).

#### 3.3. Inadequacy of specialized courses

Some participants noted that the number of hours in their specialized courses was inadequate and that the proportion of theoretical courses was much greater than their practical courses.

*“Trauma is a very broad topic including emergency scene management and different types of trauma. However, the course on trauma is provided to us in only seventeen hours; hence, our instructor has no option but to provide a summary of different aspects of trauma”* (P7).

### 4. Theory-practice gap

Most participants had faced uncertainties and confusion due to the incongruence between what they had learned in theoretical courses and what they observed during prehospital missions. They referred to a wide gap between standard guidelines and actual practice in missions. The two subcategories of this category were incongruence between the protocols and theoretical knowledge and non-adherence to care standards in prehospital settings.

#### 4.1. Incongruence between protocols and theoretical knowledge

Most participants highlighted that there was incongruence between their theoretical knowledge obtained in university settings and the available protocols for EMS delivery.

*“We had a practical course in an EMS station. We were dispatched on a mission with ambulance crew. The case was a patient with asthma. I witnessed all steps of care, but they were not congruent with what we had learned in classroom. After the mission, I asked the crew about the incongruence. They said that the mission protocols were periodically revised”* (P12).

#### 4.2. Non-adherence to care standards in prehospital setting

Most participants noted that the ambulance crew did not adhere to the standards of patient care and patient transfer during missions.

*“Because of its great importance, I learned cardiopulmonary resuscitation with all its details. However, in several missions with ambulance crew, I saw that cardiopulmonary resuscitation at emergency scenes was not performed according to the standards”* (P4).

## Discussion

This study explored EMS students’ perceptions of the challenges of EMS education. Findings showed that the four main challenges of EMS education were shortcomings of the clinical education environment, lack of qualified EMS instructors, shortcomings of the curriculum and teaching methods, and theory-practice gap.

The shortcomings of the clinical education environment were among the most important challenges of EMS education. A former study showed that there was no clear guideline for determining the time and place for EMS courses and evaluating the courses ( 14 ). Another study reported that the lack of opportunities for care delivery to patients in prehospital settings prevented the development of EMS students’ creativity and practical skills ( 15 ). Similarly, a qualitative study concluded that appropriate environment was needed for achieving the objectives of clinical courses for paramedical students ( 14 ). The Canadian Medical Association states that educational centers need to provide EMS students with appropriate clinical and prehospital environment, improve their clinical skills, train them in all clinical routines and skills, and empower them for independent practice ( 16 ). Despite the eighteen-year history of the EMS discipline in Iran, there are inadequate appropriate clinical and prehospital environments for EMS students, limited EMS stations for student training, and unclear guidelines for training students in prehospital settings; hence, the students cannot adequately experience EMS missions and EMS delivery in prehospital settings.

The findings of this study also indicated the lack of qualified EMS instructors as a main challenge of EMS education. Most EMS instructors in Iran do not have long experience of education and work in the field of EMS. A former study reported clinical instructors’ incompetence, technical skills of nursing staff, and a non-conducive clinical learning environment as the main factors affecting the clinical preparation of nursing students ( 17 ). Another study also showed limited readiness of clinical instructors as a main factor contributing to the under-preparation of EMS students. Clinical instructors’ incompetence is a significant factor contributing to the inadequate preparation of EMS students ( 18 ). The education of EMS students is among the responsibilities of medical sciences universities, particularly the faculties of nursing and allied medical sciences. In these faculties, instructors mostly hold Master’s or PhD degrees in nursing and have limited educational and work experience in the area of EMS. Therefore, they have problems in providing quality education to EMS students about triage in emergency conditions, advanced trauma, patient transfer, and EMS delivery in special conditions.

Shortcomings of the curriculum and teaching methods were the third main category of the challenges of EMS education in the present study. Despite great advances in teaching methods and education, traditional methods such as lecture are still widely used in some faculties. In traditional teaching methods, just instructors are involved in education and students are mostly passive listeners. However, education for EMS students should improve their problem solving and critical thinking skills ( 19 ). A study reported that problem-based learning was necessary for EMS students in order to improve their decision making skills ( 20 ). Another study introduced electronic learning as an interesting, flexible, and interactive method for providing realistic clinical cases ( 19 ). Electronic learning also provides opportunities for attaining learning objectives, is a source of brainstorming, and is a method for exchanging information and ideas. Electronic learning methods are effective strategies for developing education in universities and can be useful for students in settings with no in-service education or limited competent instructors ( 21 , 22 ). In medical sciences, educational curriculums are inevitably revised due to rapid global changes in healthcare delivery, technological developments, emerging diseases, patients’ expectations, and improved knowledge about human body ( 23 ). Lack of such revision can be considered as a factor contributing to the shortcomings of the curriculum for EMS students in the present study.

Theory-practice gap was the last main category of the challenges of EMS education in the present study. Many different factors can contribute to the theory-practice gap in healthcare sciences. These factors include the shift from practical training to academic education in universities ( 24 ), students’ limited knowledge ( 25 ), problems in the use of theory in practice ( 26 ), formulating theory based on idealized situations, situational considerations, students’ dependence on practical instructors, instructors’ relationships with students, and instructors’ attitudes, beliefs, experience, and qualifications ( 27 ). Theory-practice gap in the present study can also be due to the lack of appropriate clinical environments, instructors’ limited professional competence, and ineffective communication between students and instructors.

### Limitations

This study was conducted in EMS students with bachelor’s degree and in a single school of nursing in the west of Iran. Further studies on the faculty members, planners of EMS and other experts and in different contexts are needed to identify the challenges of EMS education.

## Conclusion

This study concludes that EMS students face many different challenges during their education including challenges related to their instructors, curriculum, and learning environment. Because this group of students will be at the forefront of providing medical care to critically ill patients and injured people in the future, they should receive the most appropriate theoretical and practical training before entering real environments. University authorities, health policy makers, EMS authorities and managers need to develop culturally appropriate strategies in joint committees in order to manage these challenges and promote EMS students’ learning.
